# Role of Behavioral Addictions in Predicting Reactivity in Bipolar Mood Disorder Patients

**DOI:** 10.5812/ijhrba.13909

**Published:** 2014-03-10

**Authors:** Abbas Abolghasemi, Hasan Sadeghi, Azar Kiamarsi, Moslem Abbasi

**Affiliations:** 1 Department of Psychology, University of Mohaghegh Ardabili, Ardabil, IR Iran; 2 Young Researchers Club and Elites, Islamic Azad University Branch Science and Research of Ardabil, Ardabil, IR Iran; 3 Department of Psychology, Islamic Azad University Branch Science and Research of Ardabil, Ardabil, IR Iran; 4 Department of Psychology, Faculty of Literature and Human Sciences, Salman Farsi University of Kazerun, Kazerun, IR Iran

**Keywords:** Behavior, Addictive, Interpersonal Relations, Bipolar Disorder

## Abstract

**Background:**

Behavioral addictions (BAs) can be understood as disorders characterized by repetitive occurrence of reactivity and uncontrolled behaviors. Very few studies have investigated their association with bipolar mood disorders.

**Objectives:**

The present study aimed to determine the role of behavioral addictions in predicting interpersonal behavioral addictions in bipolar mood disorder patients.

**Materials and Methods:**

This study had a cross-sectional correlation design. The statistical population was composed of all outpatients with bipolar mood disorders referring to clinical centers in Ardabil. The sample included 60 bipolar mood patients selected from patients referring to clinical centers using the available sampling method. A researcher-made behavioral addiction checklist, Interpersonal Behavioral Addictions Index, and exercise, sexual, and work addiction questionnaires, were used for data collection. The data were analyzed with a Pearson’s correlation coefficient and multivariate regression analysis.

**Results:**

The results showed a significant negative relationship between behavioral addictions and interpersonal behavioral addictions (P ≥ 0.01). Multivariate regression analysis results also showed that behavioral addictions are significant and can explain 61% of the variance of interpersonal behavioral addictions in bipolar mood patients.

**Conclusions:**

These results suggest that addictive behaviors can affect behavioral addictions in bipolar mood patients. Behavioral addictions lead to negative emotional regulation strategies and result in increased behavioral addictions in these patients. People with high levels of arousal or those who cannot control their behavioral addictions are probably more prone to addictive behaviors.

## 1. Background

Behavioral addictions (BAs) can be considered as disorders with repetitive occurrence of impulsive behaviors and specified uncontrollability, as well as psychological, cognitive, social and occupational problems, which sometimes have legal or economic consequences. The main features of these disorders include; failure to resist impulses or temptations to do dangerous things towards self or others, together with increased feelings of tension or excitement before doing these behaviors and a sense of fun while performing the desired behavior or shortly after it (1).

The DSM-IV-TR does not include a separate classification and diagnostic criteria for BAs, except for pathological gambling, which falls into the impulse control disorders category (2). The debate still continues whether BAs are associated with mood disorders (3), substance use disorders (4), impulse control disorders (5), or belong to the range of practical obsessive-compulsive disorders (6, 7). Mood disorders are among the most common psychiatric disorders, exhibiting a depressed mood in periods of depression and an elevated mood during mania periods (8). Although bipolar mood disorder (BMD) is traditionally considered to be a disease with desirable results in the long-term, a large percentage of bipolar mood patients in recent studies have reported severe periods of the condition, along with a high recurrence of residual symptoms of cognitive and functional disorders, as well as chronic psychosocial disabilities (9). Mortality rates in people with bipolar mood disorder are two to three times higher than in the general population. In total 10%-20% of BMD patients suffer from thoughts of suicide and a third of them have attempted suicide at least once (9). Thus, clinical demonstrations of BMD include a wide range symptoms from; hypomania and moderate depression, to mania or severe depression with psychotic features, and also a mixed state (9). Comorbidity between BAs and mood disorders has not been studied systematically and the results remain controversial, although studies have shown high rates of major depression and dysthymia in pathological gambling (10-12).

Reactivity refers to appraisals of the stress, coping strategies used, as well as the extent to which self-reported well-being or cortisol levels are altered by the experience of interpersonal tensions. Individuals with bipolar mood disorder often display addictive behaviors, and exhibit greater emotional reactivity compared to healthy controls, independent of current symptom levels (5).

In a study on 20 people with a compulsive buying disorder, Gruber et al. (5) reported that 50% of the subjects were affected by major depression. In another study by Lejoyeux et al. (13), compulsive buying in depressed patients was significantly associated with impulse control disorders. One study has reported the relationship between major depression or dysthymia and compulsive sexual behavior (14). A relationship has been found between Internet addiction and depression disorders in a majority of studies (15, 16). Addictive behaviors have been observed among patients with bipolar mood disorders especially during manic episodes, but it may also occur during euthymic periods or other mood phases (17, 18). In the manic state, a high level of aggression has been described, which is dependent on impulsive behavior (19, 20). In addition, a high prevalence of bipolar mood disorder comorbidity has been observed in patients with impulse control disorders (21, 22), which in turn, predict BAs in subjects. Moreover, many studies have emphasized the occurrence and continuation of addictive behaviors in bipolar mood and schizophrenia patients (22). Relationships between smoking and alcohol consumption (23), alcohol use and violence (24), being exposed to violence and alcohol use (25), and early onset of sexual contact and drug use (26), have been investigated. Blackson et al. (27) showed that emotional BAs are associated with the early onset of substance use and addictive behaviors. High BAs in bipolar mood disorders lead to the use of negative emotional regulation strategies and thus higher usage of drugs. People who cannot control their impulses are more likely to be at risk for increased substance abuse (28). In a study by Sanchez et al. (29), it was found that mood instability in bipolar mood disorder was associated with suicidal tendencies. Studies have suggested that addictive behaviors in patients with BAs are much higher than in schizophrenic or other patients (30). 

There have been a limited number of research findings on the relationship between BAs and reactivity, and the important role of BAs on reactivity in bipolar patients. The present study aimed to determine the relationship between BAs and interpersonal reactivity in bipolar patients.

## 2. Objectives

The present study aimed to determine the role of BAs in predicting interpersonal BAs in bipolar mood disorder patients.

## 3. Materials and Methods

This study had a cross-sectional correlation design. The statistical population included all outpatients with bipolar mood disorders referring to clinical centers in Ardabil from September 2012 to March 2013. The statistical sample consisted of 60 bipolar mood patients selected from patients referring to clinical centers with the convenience sampling method. In correlation studies, the sample size needs to be at least 30 subjects. But in this study a sample size of 60 was considered so that the selected sample was an actual representation of the population and had high external validity. The study used the following tools for data collection:

Checklist of behavioral addictions: This study used a researcher-made checklist of BAs (eating addiction, alcohol addiction, TV and mobile addictions). The formal validity of the questionnaire was confirmed by various field experts. The formal validity of the questionnaire was confirmed by five experts. After gathering their opinions, the final checklist was developed with 32 questions. Cronbach's alpha coefficient was 0.78 in the present study.

Sexual Addiction Screening Test: This scale was developed by Carnes (31). This questionnaire was designed to help assess sexual impulsivity behaviors which may indicate sexual addiction. This scale has 25 items with Yes or No answers. Carnes (31) reported Cronbach's alpha reliability coefficients for this scale to be 0.77 and 0.76, respectively. In the present study, the Cronbach's alpha coefficient obtained was 0.80. 

Work Addiction Risk Test: The scale was developed by Robinson and Post (32) and contains 25 items. In that study the reported Cronbach's alpha reliability coefficients for this index were 0.67 and 0.76, respectively. In the present study, the Cronbach's alpha coefficient obtained was 0.78. 

### 3.1. Exercise Addiction Inventory

The short form of the exercise addiction inventory was developed by Griffiths (33). This form consists of six statements based on a modified version of behavioral addiction components. Each statement is scored based on a 5-point Likert scale, from strongly disagree (1) to strongly agree (5). Griffiths (33) reported Cronbach's alpha reliability coefficients for this index at 0.87 and 0.96, respectively. In the present study, the Cronbach's alpha coefficient obtained was 0.84.

Interpersonal behavioral addictions index: The Interpersonal BAs index was designed to measure interpersonal behaviors (24). This questionnaire had 28 items, each of which can be responded based on a likert scale (‘it does not describe me very well’ to ‘it describes me very well’). Cronbach’s alpha coefficient for this index ranges from 0.75 to 0.82 (24). Cronbach’s alpha coefficient and test-retest reliability coefficient of drug addicts obtained were 0.77 and 0.76, respectively. In addition, in one study (25) it was found that there was a significant difference between drug abusers and normal individuals in terms of the interpersonal behavior scale (P < 0.01).

### 3.2. Procedure

After identifying patients with bipolar mood disorders in the clinical centers, they were told about the research objectives. Then, they were provided with the research tests and were asked to express their opinions carefully. Data were collected on an individual basis at the associated center. The collected data were then analyzed with a Pearson’s correlation coefficient and stepwise multiple regression. 

## 4. Results

A total of 60% of the participants in this study were male and 40% were female. Means and standard deviations of educational status for men and women participating in the study were as follows: 33.3% under diploma, 30% diploma, 30% associate degree and 6.7% bachelor. There were 40% of subjects who were self-employed, 28.3% state workers and 26.7% housewives.

**Table 1.** Mean and Standard Deviation of BAs and Interpersonal Reactivity in Bipolar Mood Patients, and the Correlation Between These Variables^a^

	Mean ± SD	Behavioural Addictions (BAs)	Sig
**Mobile phone addiction**	26.23 ± 3.78	-0.55	0.001
**TV addiction**	13.23 ± 2.63	-0.57	0.001
**Drug addiction**	23.75 ± 3.79	-0.69	0.001
**Alcohol addiction**	26.23 ± 3.88	-0.53	0.001
**Eating addiction**	13.23 ± 2.63	-0.57	0.001
**Exercise addiction**	21.97 ± 3.57	-0.53	0.001
**Compulsive buying**	24.55 ± 3.23	-0.64	0.001
**Work addiction**	16.08 ± 4.15	-0.52	0.001
**Bas**	39.72 ± 9.71	-	

^a^ P ≤ 0.01.

Table 1 shows the means and standard deviations for scores of BAs in bipolar patients. The results also showed that mobile phone addiction, TV addiction, alcohol addiction, eating addiction, exercise addiction, compulsive buying and work addiction are negatively associated with interpersonal reactivity (P ≤ 0.01).

**Table 2.** Results of Multivariate Regression Analysis for Predicting Reactivity Through BAs^a^

Model	R	R^2^	Non-Standard Coefficients		β	*t*	p
			B	SD Error			
**Constant**	-	-	98.43	6.57	-	14.99	0.001
**Drug addiction**	0.686	0.471	- 1.759	0.245	- 0686	- 7.19	0.001
**Compulsive buying**	0.760	0.577	- 1.149	0.304	- 0.383	- 3.78	0.001
**Eating addiction **	0.782	0.612	- 0.833	0.373	- 0.266	- 2.23	0.01

^a^ Abbreviation: BAs, behavioral addiction

To determine the strongest reactivity predictor in bipolar mood patients through BAs, the stepwise multiple regression analysis method was used. According to Table 2, the observed F-value was significant (P ≤ 0.01) and 61% of the variance of interpersonal reactivity in bipolar mood patients was explained by BAs. Given the beta values, drug addiction (Beta = -0.686), compulsive buying addiction (Beta = -0.383) and eating addiction (Beta = -0.266) are the strongest interpersonal reactivity predictors in bipolar mood patients.

## 5. Discussion

The present study aimed to investigate the role of BAs in predicting interpersonal reactivity in bipolar mood disorder patients. The results showed a negative relationship between BAs and interpersonal reactivity in bipolar mood disorders, which is consistent with previous studies (18, 34). Severe behavioral tendencies can be considered as disorders and the main characteristic is uncontrolled repetitive occurrence of a behavior that leads to psychological, social and occupational problems and sometimes legal and economic consequences. In explaining this problem, one can say that addiction caused by either psychoactive drugs or specific behaviors, involve common neurobiological mechanisms through the activation of reward pathways and multiple interference of neurological neurotransmitter systems (dopaminergic at the first stage, endogenous opioid and serotonergic at the second stage) (35), and also clinical demonstrations, including mood changes, so that the addiction process can be considered as unitary (36-38).

According to a study by Nicola et al. (39), bipolar mood patients obtained high scores in BAs, such as; pathological gambling, compulsive buying and sexual behavior, compared with a control group. These results indicated that BAs in bipolar mood patients are caused by mood instability. The results also showed that, like drug addicts, these subjects use addictive behaviors to reduce the activation caused by mood instability and their personality immaturity in general. In patients with bipolar mood disorders, BAs have been reported with greater personality immaturity and lower levels of self-assertion when compared to people without BAs (39). In addition, patients with BAs have more personality disorders compared with others. This result is consistent with other studies (25, 40). Based on the results of this study, it can be said that bipolar mood disorders are related to an inaccurate and overly negative interpretation of social situations, and these erroneous interpretations of ambiguous situations affect decision-making, BAs and the judgments of people with bipolar mood disorder. In general, one can say that inaccurate judgments and negative interpretations of social situations, such as public speaking and interacting with people, can reduce interpersonal reactivity.

The results of a multivariate regression analysis showed that BAs explained 61% of reactivity in bipolar mood patients, among which; drug addiction, compulsive buying addiction and eating addiction, had significant power for reactivity prediction in bipolar mood patients. This result showed that 39% of variance and residual factors are explained by other variables affecting reactivity. Moreover, due to a lack of corresponding results in previous studies, it can be inferred that the effect of addictive behaviors on the interpersonal reactivity of bipolar mood patients is significant. However, future studies are required to clarify this point.

These observations need to be replicated in a larger population, as further studies are necessary in order to understand the biological aspects of personality that contribute to relapses and the severity of addiction. Limitations of this research include; lack of reliability in the addicted persons responses, study participants limited to Ardabil City, failing to control the type of bipolar mood disorder, and consumption rates and drugs used, are factors which should make us cautious when generalizing the results. It is suggested that future studies are repeated on larger samples. It is also suggested that these results are used to prevent the recurrence and severity of bipolar mood disorders. More reliable results could be gained by studying individuals who have not yet started using methadone. It is also suggested that similar research be conducted with female narcotic drug addicts and addicts of different ages. Finally, morphine addicts may be trained to learn self-control and emotion regulation skills in the form of treatment projects.
